# Clustered Cases of *Rickettsia sibirica mongolitimonae* Infection, France

**DOI:** 10.3201/eid1902.120863

**Published:** 2013-02

**Authors:** Sophie Edouard, Philippe Parola, Cristina Socolovschi, Bernard Davoust, Bernard La Scola, Didier Raoult

**Affiliations:** Author affiliation: Aix-Marseille Université, Marseille, France

**Keywords:** Rickettsia sibirica mongolitimonae, Rhipicephalus pusillus, clustered cases, bacteria, rickettsia, ticks, cat, eschar, lymphangitis-associated rickettsiosis, France

**To the Editor:** *Rickettsia sibirica mongolitimonae,* a member of the tick-borne spotted fever group (SFG) of rickettsia, was first isolated from *Hyalomma asiaticum* ticks in China (*1*). The first human case was described in France in 1996, and 7 new cases were described in 2005 (*1*). This rickettsiosis was named lymphangitis-associated rickettsiosis because lymphangitis was observed in 50% of the patients (*1*). Only 17 cases have been reported, for which 7 patients had lymphangitis, and 13 had inoculation eschars, including 2 patients with 2 eschars (*1*,*2*). We report a cluster of cases of *R. sibirica mongolitimonae* infection.

Patient 1, a 73-year-old man in France, had fever, rash, lymphadenopathies, and an axillary inoculation eschar in February 2011. A diagnosis of lymphangitis-associated rickettsiosis was suspected because of the season (most cases occur in spring in France) and clinical manifestations. The patient was confined to bed for several weeks after surgical placement of a knee prosthesis when his disease occurred; the domestic cat was suspected to have introduced ticks into the home.

In April, his wife (67 years of age) (patient 2) became febrile, had popliteal lymphadenopathies associated with lymphangitis, and had an eschar on the leg from which a swab specimen was obtained. Patient 3 was their neighbor; he had the same symptoms in March 2011 but samples were not collected from him.

None of patients reported tick bites, but they were in regular contact with animals, including a cat, a dog, horses, and birds. Both patients who lived with the cat reported that it would return home with ticks. Infections in these patients were successfully treated with doxycycline.

An immunofluorescence assay for antibodies against SFG antigens showed IgG/IgM titers of 128/0 for patient 1 and 64/16 for patient 2 (*3*). DNA was extracted from the skin swab specimen of patient 2 by using the QIAamp Mini Kit (QIAGEN, Hilden, Germany). A fragment of the citrate synthase gene of *Rickettsia* spp. was amplified by PCR and sequenced. The sequence show 99.7% homology with that of the same gene sequence of *R. sibirica mongolitimonae* in GenBank (accession no. DQ423370) (*4*).

Ticks were collected from the property of the 2 patients: from the garden by flagging vegetation (*3*), from animals, and near the cat litter (Table). SFG rickettsiae were detected by specific quantitative PCR. Species identification was confirmed by specific quantitative PCR for *R. massiliae* and sequencing of outer membrane protein A gene for others species (*5*). A negative control (sterile water) and positive control (DNA from *R. montanensis* or *R. massiliae*) were included in each PCR.

**Table:** Ticks collected from property of 2 patients infected with *Rickettsia sibirica mongolitimonae*, France, 2011*

Location of tick collection	No. ticks collected	Tick species identification†	No. ticks (*Rickettsia* species)	
			Harboring rickettsial DNA	From which rickettsiae were cultured
Garden	2	ND	0	0
Dog	21	*Rhipicephalus sanguineus*	2 (*R. massiliae*)	0
Cat litter	7	ND	1 (*R. massiliae*)	1 (*R. massiliae*)
Cat	9	*Rh. pusillus*	1 (*R. sibirica mongolitimonae*)	1 (*R. sibirica mongolitimonae*)
Scrub land	5	ND	0	0

*ND, not done. †Based on 12S rRNA gene. All adult ticks were morphologically identified as *Rh. sanguineus*.

Ticks were morphologically identified as adult *Rhipicephalus sanguineus*. Molecular identification of these ticks harboring rickettsiae was performed by amplification of the 12S rRNA gene. DNA from *R. massiliae* was found in 3 ticks collected from the dog and near the cat litter morphologically identified as *Rh. sanguineus*. This DNA showed 98% homology with the sequence in GenBank (accession no. AY559843). *R. sibirica mongolitimonae* with 99.8% homology for the outer membrane protein gene sequence in GenBank (accession no. DQ097082) was isolated from 1 tick collected from the cat. This tick was identified as *Rh. pusillus* and showed 99.7% homology with the sequence in GenBank (accession no. FJ536547). *R. massiliae* was cultured from an *Rh. sanguineus* tick, and *R. sibirica mongolitimonae* was cultured from an *Rh. pusillus* tick.

A cluster of 1 documented case and 2 probable cases of lymphangitis-associated rickettsiosis in southern France was linked to a cat and *Rh. pusillus* ticks. Infection with *R. massiliae* for the 2 probable case-patients was unlikely because clinical findings were typical of lymphangitis-associated rickettsiosis, and most cases of rickettsioses in southern France in the spring are caused by *R. sibirica mongolitimonae*. Clustered cases of SFG rickettsiae infection have been reported in Europe, including southern France (*3*,*6*). In 2007, *R. conorii* and *R. massiliae* infections in humans were reported (*3*). In 2010, cases for which we were unable to discriminate between *R. conorii* and *R. massiliae* infections occurred in a family (*6*). In these 2 studies, clustered cases of SFG rickettsiosis involved *Rh. sanguineus* ticks. Clustered cases appeared to be related to an increase in aggressiveness of ticks toward humans during warmer periods (*3*). In our study, no correlation was identified with warmer weather.

*R. sibirica mongolitimonae* is most frequently associated with *Hyalomma* spp. ticks (*1*,*2*,*4*). However, 1 case of infection with this bacterium was associated with *Rh. pusillus* ticks collected in Portugal (*7*); DNA from this bacteria was also identified in an *Rh. pusillus* tick collected from a mongoose. The European wild rabbit is the primary host of *Rh. pusillus* ticks. However, these ticks have been found on wild carnivorous animals, dogs, and domestic cats (*8*); these ticks can bite humans (*8*). Moreover, *R. massiliae* and *R. sibirica mongolitimonae* were found in *Rh. pusillus* ticks from Spain (*9*), and SFG rickettsiae were found in ticks from Sardinia (*10*). Therefore, *Rh. pusillus* ticks appear to be an emerging vector for *R. sibirica mongolitimonae* in Europe.
